# Unlocking the genetic potential of cauliflower: the ‘lucky’ cultivar and *Bacillus subtilis* synergy for superior productivity and bioactive enrichment

**DOI:** 10.3389/fpls.2026.1829722

**Published:** 2026-05-07

**Authors:** Mehboob Alam, Ibadullah Jan, Baokun Lei, Yongbo Xu, Muhammad Sajid, Bo Fan

**Affiliations:** 1 Department of Horticulture, Faculty of Crop Production Sciences, The University of Agriculture, Peshawar, Pakistan; 2 Department of Agriculture, The University of Swabi, Swabi, Pakistan; 3 Agricultural Environment and Resources Institute, Yunnan Academy of Agricultural Sciences, Kunming, China; 4 Key Laboratory for Improving Quality and Productivity of Arable Land of Yunnan Province, College of Resources and Environment, Yunnan Agricultural University, Kunming, China

**Keywords:** *Bacillus subtilis*, cauliflower, cultivar, curd, yield

## Abstract

The synergistic interplay between plant growth-promoting bacteria (PGPB) and elite cauliflower cultivars presents a sustainable approach to enhance crop productivity and nutritional quality. This study evaluated the interactive effects of three *Bacillus* strains (*B. subtilis, B. thuringiensis, and B. pumilus*) on vegetative growth, reproductive yield, and bioactive compound accumulation in three cauliflower cultivars (‘Lucky’, ‘White Beauty’, and ‘Snow Drift’). Significant genetic variability was observed among cultivars, with ‘Lucky’ consistently outperforming others across all parameters, exhibiting maximum plant height, leaf area, chlorophyll content, curd dimensions, and yield attributes. Among PGPB strains, *B. subtilis* proved to be the most effective, significantly enhancing vegetative development, reproductive performance, and bioactive compound accumulation, followed by *B. thuringiensis*. To our knowledge, this study provides the first empirical evidence that cultivar-specific compatibility with *B. subtilis* amplifies flavonoid accumulation by 46% and curd dry weight by 33.3% in cauliflower, suggesting that this synergistic pair represents a promising strategy for sustainable vegetable production. Novel cultivar-specific synergistic interactions were uncovered: the combination of ‘Lucky’ with *B. subtilis* produced the highest total yield and maximum anthocyanin accumulation, while ‘White Beauty’ paired with *B. subtilis* yielded the highest phenolic content, and ‘Lucky’ with *B. thuringiensis* generated maximum leaf area. Additionally, ‘White Beauty’ treated with *B. subtilis* exhibited superior chlorophyll content, indicating enhanced photosynthetic capacity. The significant cultivar × PGPB interactions for total yield, phenolics, and anthocyanins underscore the critical importance of strain-specific compatibility for maximizing both productivity and nutraceutical value. These findings provide compelling evidence that strategic integration of genetically superior cultivars with compatible PGPB strains—particularly the ‘Lucky’ × *B. subtilis* combination—offers a offers a viable and effective for sustainable cauliflower production, has practical implications for developing cultivar-specific bio-inoculant strategies that simultaneously enhance food security and nutritional quality in changing climates.

## 1 Introduction

Cauliflower (*Brassica oleracea* var. botrytis L.), a member of the Brassicaceae family, is a globally significant vegetable crop prized for its edible white curd (Kushwaha et al., 2026). Chemical fertilizers have historically contributed to increased crop productivity; however, their excessive use has also been associated with environmental degradation, soil quality decline, and disruption of beneficial microbial communities. In this context, bio-inoculants have emerged as a promising component of more sustainable crop management strategies (Topa et al., 2025).

PGPB are free-living, root-colonizing, or endophytic microorganisms that exert beneficial effects on plants through a variety of direct and indirect mechanisms (Zambrano-Mendoza et al., 2021). The concept of PGPB, first explored in the 1950s, has since evolved into a major field of biotechnological research with applications spanning agriculture, horticulture, and environmental protection (Zahir et al., 2004; Wang et al., 2025). PGPB represent a diverse array of bacterial classes, including Actinobacteria, Firmicutes, and Proteobacteria, and genera such as *Pseudomonas*, *Azospirillum*, and *Bacillus* (Tilak et al., 2005; Su et al., 2023). Their modes of action include enhanced nutrient acquisition (Dey et al., 2004; Ilyas et al., 2023; Zhao et al., 2025), synthesis of plant growth regulators (Glick et al., 2007; Sun et al., 2025), ACC deaminase activity, and suppression of phytopathogens (Zeng et al., 2020).

Bacteria of the genus *Bacillus* are spore-forming and hold immense potential due to their resilience and multifunctional traits. Their ability to form durable endospores allows for the formulation of stable and long-lasting bio-inoculants. *B. subtilis* produces ACC deaminase, helping to modulate stress-induced ethylene levels and maintain normal root and shoot growth under adverse conditions (Glick et al., 2007). Its colonization of the rhizosphere and root surfaces also involves the secretion of exopolysaccharides and siderophores, which improve water and nutrient translocation and create a protective barrier against pathogens (Radhakrishnan et al., 2017). This symbiotic interaction is mutually beneficial, as plants exude up to 30% of their photosynthetically fixed carbon as nutrients into the rhizosphere, fueling bacterial activity (Allard et al., 2016). *B. pumilus* contributes to plant growth through its capacity for atmospheric nitrogen fixation, mediated by the nitrogenase enzyme (Masood et al., 2020). It has been shown to enhance plant development under both normal and saline conditions (Upadhyay et al., 2019; Shahzad et al., 2021; Kumar et al., 2021). However, research also presents a complex picture, with some studies indicating that its growth-promoting effects may be context-dependent and not beneficial under all abiotic stresses, such as caesium toxicity in *Brassica* species (Aung et al., 2015), highlighting the need for crop- and condition-specific evaluations (Khan et al., 2016). *B. thuringiensis* produces crystalline (Cry) proteins (delta-endotoxins) during sporulation that are highly specific to the larvae of various insect pests, such as *Spodoptera exigua* (beet armyworm), a common threat to cauliflower and other cole crops (Schnepf et al., 1998; Dingha et al., 2004; Hassan et al., 2023). Crucially, these Cry proteins are non-toxic to humans, mammals, and beneficial insects, making them an environmentally safe component of integrated pest management. Beyond its role as a biopesticide, it also exhibits direct PGPB traits, including phosphate solubilization, IAA production, and siderophore synthesis, thereby contributing to plant nutrition and growth (Jouzani et al., 2017).

The pressing need for sustainable alternatives to chemical inputs in vegetable production provides a strong rationale for investigating PGPB in cauliflower. While the individual benefits of these bacterial strains are well-documented in other crops such as rice, wheat, and maize (Zambrano-Mendoza et al., 2021), and previous research has documented PGPR-mediated growth promotion in cauliflower (Gaffar and Kushwaha, 1994; Jat and Bhardwaj, 2005; Metz, 2003; Peptech Biosciences, 2026; Raddadi et al., 2007; Singh et al., 2024; Trasmundi et al., 2025), their effects on the growth, yield, and quality of cauliflower cultivars remain underexplored. Furthermore, although cultivar-specific PGPR compatibility has been demonstrated in other crops (Schenk et al., 2024), no study has systematically evaluated the following hypothesis: First, cauliflower cultivars differ significantly in their growth, yield, and quality parameters due to genetic variability. Second, the three Bacillus strains (*B. subtilis, B. thuringiensis, and B. pumilus*) differ in their ability to promote growth, enhance yield, and improve bioactive compound accumulation. Third, a significant cultivar × strain interaction exists, meaning the effectiveness of a given strain depends on the specific cultivar to which it is applied. Fourth, different cultivar-strain combinations optimize different agronomic and quality traits, including yield, phenolics, anthocyanins, and flavonoids. Fifth, the combination of ‘Lucky’ with *B. subtilis* is predicted to yield the highest overall performance.

Therefore, this study was designed to evaluate the effect of selected PGPB strains (*B. thuringiensis*, *B. subtilis*, and *B. pumilus*) on the growth, yield, and quality parameters of cauliflower cultivars.

## 2 Materials and methods

### 2.1 Study site and experimental conditions

A field experiment was conducted to evaluate the interactive effects of PGPB strains on the performance of different cauliflower cultivars. The experiment was conducted under a shade house environment at the Ornamental Horticulture Nursery of The University of Agriculture, Peshawar (34° 1’ 50.9” N, 71° 25’ 5.0” E). The soil was sandy loam in texture with pH 7.7, electrical conductivity (EC) 0.54 dS m^-1^, and organic matter 0.8%. Available nutrient levels were 185 mg kg^-1^ N, 12.5 mg kg^-1^ P, and 145 mg kg^-1^ K. During the growing season, the mean air temperature ranged from 12 to 22 °C, relative humidity averaged 75 ± 5%, and average solar radiation was 907.4 W m^-2^ (measured with a TES 1333 solar power meter, PR China).

### 2.2 Experimental design and treatments

The experiment was laid out in a Randomized Complete Block Design (RCBD) with a two-factor factorial arrangement. Factor A comprised three commercially cultivated cauliflower cultivars: ‘Snow Drift’ (C1), ‘Lucky’ (C2), and ‘White Beauty’ (C3). Factor B consisted of four inoculation treatments, including an untreated control and three Bacillus species: T0 = Control (no bacteria), T1 = *B. subtilis* (strain 21332), T2 = *B. pumilus* (strain C-ISSK-8), and T3 = *B. thuringiensis* (strain C-2PMW-8). There were three blocks in the experiment. Each block contained 12 treatment combinations, and each treatment combination was randomly assigned to one experimental plot per block.

### 2.3 Field preparation and crop husbandry

The experimental field was prepared by deep plowing with a cultivator, followed by planking to achieve a fine tilth. The field was meticulously cleared of weeds, stones, and crop residues from the previous harvest. Well-decomposed farmyard manure (FYM) was incorporated into the soil at a rate of 25 t ha^-1^, 20 days prior to transplanting, to improve soil structure and fertility. Uniform, healthy, and disease-free cauliflower seedlings, approximately 12–15 cm in height, were procured from a commercial nursery and transplanted on October 24th, 2023. A basal dose of NPK fertilizers was applied at the time of transplanting at a rate of 200:75:75 kg ha^-1^, using urea, single superphosphate, and sulfate of potash as sources (Krishnakumar et al., 2005). Seedlings were planted on raised beds at a spacing of 60 cm between rows and 45 cm between plants. Standard agronomic practices, including regular weeding, hoeing, and irrigation, were performed uniformly across all plots throughout the growing season to ensure optimal crop growth and to minimize biotic and abiotic stress.

### 2.4 Bacterial strains, inoculum preparation, and application

The three *Bacillus species* used in this study were procured from the stock culture collection of the Institute of Biotechnology and Genetic Engineering (IBGE), The University of Agriculture, Peshawar. The specific strains utilized were *B. pumilus* (C-ISSK-8), *B. thuringiensis* (C-2PMW-8), and *B. subtilis* (21332).

For inoculum preparation, the bacterial strains were first reactivated by streaking onto petri plates containing nutrient agar (NA) medium (32 g L^−1^) and incubated at 27 °C for 48 hours. A single colony from each pure culture was then transferred aseptically into 500 mL Erlenmeyer flasks containing 250 mL of sterile nutrient broth (NB) medium (16 g L^−1^). The flasks were incubated aerobically in an orbital shaking incubator at 150 rpm and 27 °C for 48 hours to allow for bacterial multiplication.

Following incubation, the optical density (OD) of each bacterial suspension was measured at 660 nm using a spectrophotometer (R&M marketing S-200, England). Preliminary calibration experiments established that an OD_660_ of 0.100 corresponds to approximately 10^6^ CFU mL^−1^ for all three Bacillus strains. This calibration was performed by preparing serial dilutions of each culture, plating onto nutrient agar, and counting colonies after 48 hours of incubation at 27 °C.

Before each application, the final concentration (≈10^6^ CFU mL^−1^) was verified by plate counting on nutrient agar. The bacterial suspensions were then adjusted to the target concentration by diluting with sterile distilled water as needed.

The bacterial suspensions were applied as a foliar spray to the cauliflower plants. The first application was carried out 15 days after transplanting (DAT), and a second application was performed 45 days after transplanting. All applications were performed in the morning (8:00–10:00 AM) to minimize evaporation. A spray volume of 250 mL per plot (equivalent to approximately 500 L ha^−1^) was used, which was sufficient to ensure uniform coverage of the foliage without run-off. No surfactant or adjuvant was added to the bacterial suspensions. Control plots were sprayed with an equivalent volume of sterile distilled water under the same conditions.

### 2.5 Parameters studied

For each experimental unit (plot), measurements from five randomly selected plants were averaged to obtain a single value per plot. These plot-level means were then used in the statistical analysis, with three blocks providing three replicate values per treatment combination. Therefore, all reported means are the averages of three replications (blocks), not of individual plants. Standard deviations were calculated from the three block means (n = 3).

#### 2.5.1 Measurement of growth and development parameters

Plant height, number of leaves per plant, leaf area, chlorophyll content, and survival percentage were recorded according to the methods described by Al-Zuhairy and Al-Hamdany (2017); Zalud (1999), and Wenneck et al. (2021), respectively. Survival percentage was calculated throughout the growing season using the following formula:


$$Survival\;percentage=\frac{Number\;of\;plants\;survived\;\;\;\;}{Total\;number\;of\;Plants\;transplanted}\;\times \;100$$

#### 2.5.2 Reproductive and yield parameters

Data were also recorded on reproductive parameters like days to curd appearance, days to curd harvest, curd diameter (cm), curd fresh weight (kg), curd dry weight (kg), and total curd yield (tons ha^-1^) as described by Al-Zuhairy and Al-Hamdany (2017).

#### 2.5.3 Determination of biochemical and quality

Fresh curd samples were used to evaluate the nutritional and nutraceutical quality of cauliflower as influenced by bacterial inoculation. All biochemical analyses were performed on a fresh weight (FW) basis, and results were expressed as mg g^−1^ fresh weight.

Sample preparation: Fresh curd (2 g) was homogenized with 10 mL of 80% (v/v) methanol (1:10 w/v) using a mortar and pestle. The homogenate was incubated at 25 °C for 2 hours with gentle shaking (120 rpm) to allow complete extraction. The mixture was then centrifuged at 4000 rpm for 10 minutes at room temperature, and the supernatant was collected for subsequent analyses.

Total phenolic content was determined according to Önder et al. (2020). Total flavonoid content was measured as described by Sultana et al. (2024). Anthocyanin content was measured according to Ali et al. (2023). Total carotenoids were determined using the procedure described by Wenneck et al. (2021).

### 2.6 Statistical analysis

All collected data were subjected to analysis of variance (ANOVA) using Statistik 8.1 software (Statistix^®^; Analytical Software Inc., Tallahassee, FL, USA). The full ANOVA model included the main effects of cultivar (C), bacterial treatment (B), and their interaction (C × B).

Assumption testing: Prior to ANOVA, the assumptions of normality of residuals and homogeneity of variances were tested using the Shapiro-Wilk test and Levene’s test, respectively. Both assumptions were satisfied (p > 0.05 for all cases).

When significant differences were detected in the ANOVA (p ≤ 0.05), treatment means were compared using the Least Significant Difference (LSD) test at the 5% probability level (Steel and Torrie, 1997). Exact p-values are reported in the text and tables where applicable.

## 3 Results

### 3.1 Vegetative growth

Vegetative growth parameters varied significantly among cauliflower cultivars and in response to PGPB inoculation (**Table 1**). Cultivar × PGPB interactions were non-significant for plant height, number of leaves per plant, and survival percentage, but were significant for leaf area and leaf chlorophyll content (**Table 1**). Among the three cultivars, ‘Lucky’ showed the highest mean values for plant height (47.1 cm), number of leaves per plant (24.6), single leaf area (1024.9 cm²), chlorophyll content (54.0 SPAD), and survival percentage (84%), followed by ‘White Beauty’, while ‘Snow Drift’ consistently showed the lowest values for all parameters. PGPB-promoting bacterial inoculation had a pronounced effect on cauliflower plant stature compared to the untreated control. PGPB inoculation significantly affected vegetative growth traits. Among the tested strains, *B. subtilis* was associated with the highest mean values for plant height (47.9 cm), number of leaves per plant (24.5), leaf area (1077.4 cm²), chlorophyll content (54.0 SPAD), and survival rate (83%) (**Table 1**).

**Table 1:** Effect of PGPB on plant height, number of leaves plant^-1^, leaf area, chlorophyll content and survival percentage of cauliflower cultivars.

Cultivar (C)	Plant height (cm)	Number of leaves plant^-1^	Leaf area (cm^2^)	Chlorophyll (SPAD)	Survival (%)
Snow Drift	39.1 ± 5.9c	22.0 ± 1.3c	879.0 ± 119.6c	47.4 ± 1.8b	78 ± 2.5c
Lucky	47.1 ± 4.1a	24.6 ± 1.5a	1024.9 ± 55.9a	54.0 ± 2.4a	84 ± 2.2a
White Beauty	43.3 ± 4.1b	23.4 ± 1.3b	964.9 ± 118.5b	52.0 ± 3.8a	81 ± 1.8b
**LSD _(p≤ 0.01)_**	**2.40**	**0.80**	**66.20**	**1.90**	**1.80**
*Bacillus* spp. (BS)					
B0	38.4 ± 4.6c	21.8 ± 1.0c	831.1 ± 95.3c	47.6 ± 2.1c	78 ± 2.6c
BP	42.0 ± 5.6b	22.9 ± 1.6b	928.8 ± 114.2b	51.2 ± 2.4b	81 ± 2.9b
BT	44.5 ± 5.1b	24.1 ± 1.2a	987.7 ± 49.2b	51.9 ± 3.4ab	82 ± 3.5b
BS	47.9 ± 4.6a	24.5 ± 1.0a	1077.4 ± 43.6a	54.0 ± 4.3a	83 ± 1.9a
**LSD _(p≤ 0.01)_**	**2.80**	**0.90**	**76.40**	**2.20**	**2.10**
**C × BS**	**NS**	**NS**	* **Figure 1**	* **Figure 1**	**NS**

B0, Distilled water; BP, *B. pumilus*; BT, *B. thuringiensis*; BS, *B. subtilis*; Values are means of three replications (blocks) ± standard deviation (SD). Within each plot, five individual plants were sampled and averaged to obtain one value per plot before statistical analysis. Means followed by different lowercase letters within a column are significantly different at p ≤ 0.05 according to the Least Significant Difference (LSD) test. NS, non-significant; *significant at p ≤ 0.05.

Bold values in the LSD row are formatted for visual emphasis only and do not represent a statistical comparison.

**Figure 1:**
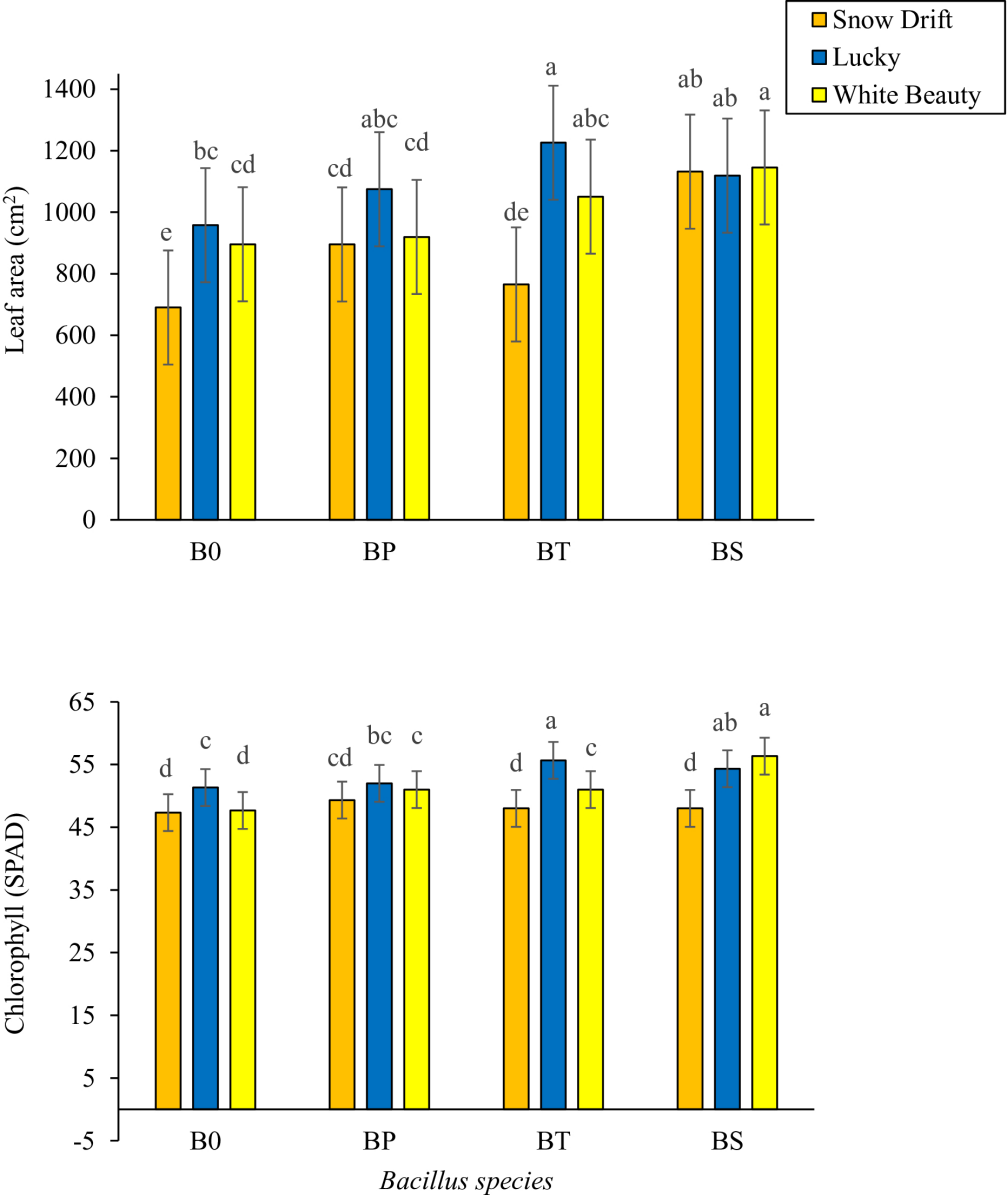
Effects of *Bacillus species* (B0, Distilled water; BP, *Bacillus pumilus*; BT, *Bacillus thuringiensis*; BS, *Bacillus subtilis*) on leaf area and leaf chlorophyll content of cauliflower cultivars (Snow drift, Lucky and White beauty). Bars with different lowercase letters indicate significant differences at p ≤ 0.05 (LSD test). Error bars represent standard error of the mean (SEM, n=3).

The cultivar × PGPB interaction significantly affected leaf area and chlorophyll content. The highest leaf area (1226.00 cm²) was observed in cultivar ‘Lucky’ treated with *B. thuringiensis*, followed by cultivar ‘White Beauty’ (1145.70 cm²) inoculated with *B. subtilis*; these two were statistically similar. The control treatment (distilled water) showed the lowest mean values for leaf area. The highest chlorophyll content (56.33 SPAD) was measured in cultivar ‘White Beauty’ treated with *B. subtilis*, followed by cultivar ‘Lucky’ (55.67 SPAD) inoculated with *B. thuringiensis*; these two were also statistically non-significant (**Figure 1**). These results suggest that, under the conditions of this study, ‘Lucky’ showed superior vegetative growth compared to the other tested cultivars, and *B. subtilis* was associated with enhanced vegetative development across all cultivars.

### 3.2 Reproductive and yield parameters

**Figure 2:**
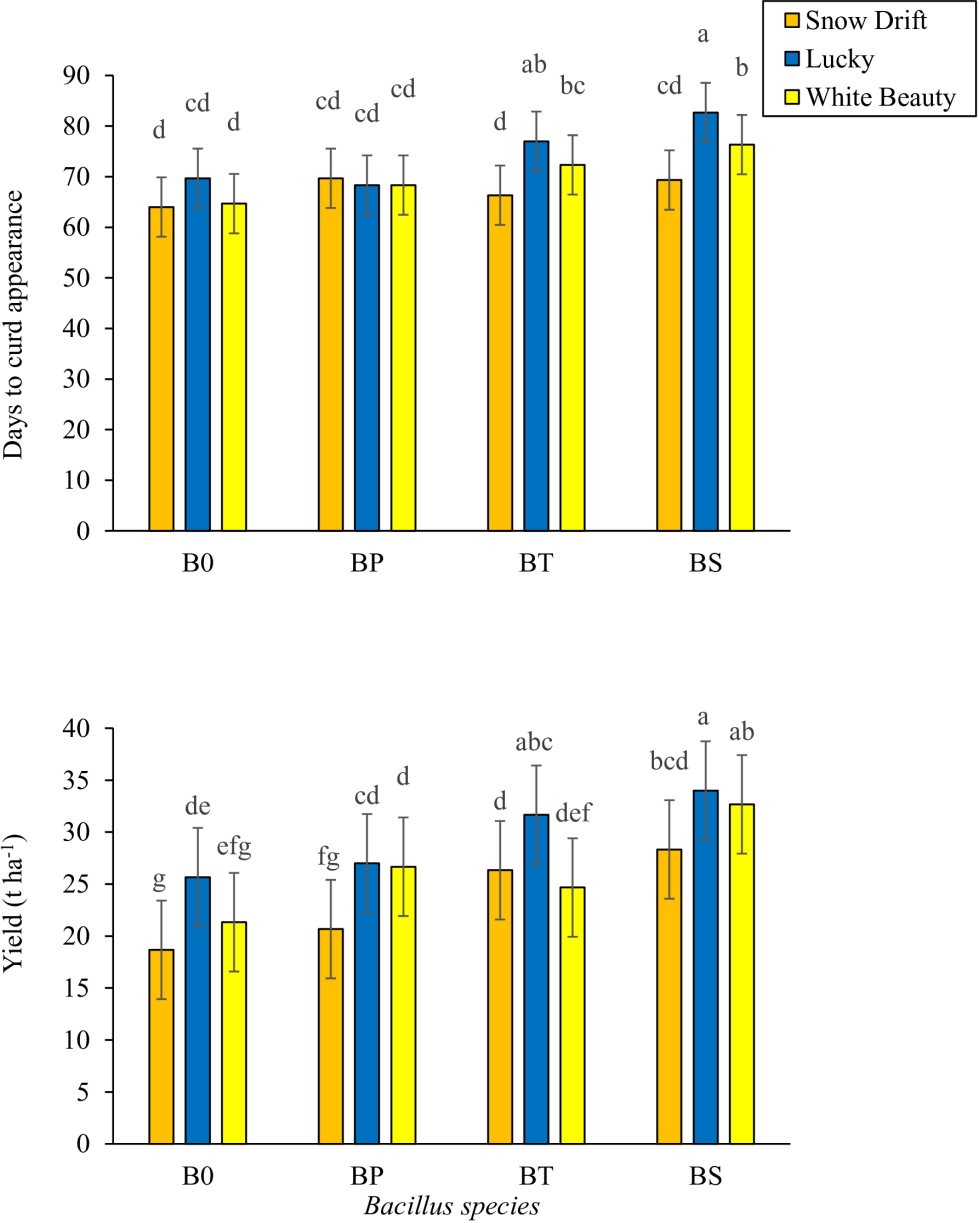
Effects of *Bacillus species* (B0, Distilled water; BP, *Bacillus pumilus*; BT, *Bacillus thuringiensis*; BS, *Bacillus subtilis*) on days to curd appearance and yield of cauliflower cultivars (Snow drift, Lucky and White beauty). Bars with different lowercase letters indicate significant differences at p ≤ 0.05 (LSD test). Error bars represent standard error of the mean (SEM, n=3).

Significant variation in reproductive and yield parameters was observed among cauliflower cultivars and in response to PGPB inoculation, with non-significant cultivar × PGPB interactions for most traits except days to curd appearance and total yield, which exhibited a significant interaction (**Table 2**, **Figure 2**). Among cultivars, ‘Lucky’ showed the highest mean values for curd diameter (31.0 cm), curd fresh weight (1.11 kg), curd dry weight (0.11 kg), and total yield (29.8 t ha^−1^), followed by ‘White Beauty’. ‘Lucky’ also required the longest period for curd appearance (73.6 days) and harvest (108.5 days). In contrast, ‘Snow Drift’ showed the earliest maturity (66.6 days to curd appearance; 102.0 days to harvest) but the lowest values for curd dimensions and yield. Across all cultivars, *B. subtilis* was associated with the highest mean values for curd diameter (28.7 cm), curd fresh weight (1.15 kg), curd dry weight (0.12 kg, representing a 33.3% increase over control), and total yield (31.0 t ha^−1^), followed by *B. thuringiensis*. *B. subtilis* was also associated with a longer period to curd appearance (75.6 days) and harvest (109.8 days) compared to the control. Control plots showed the earliest maturity but the lowest yield attributes (**Table 2**). The interactive effect of cauliflower cultivars and *Bacillus species* was also significant regarding days to curd appearance. The highest days to curd appearance (82.67) were taken by cultivar ‘Lucky’ inoculated with *B. subtilis*, followed by the same cultivar (77.00 days) treated with *B. thuringiensis*. The significant cultivar × PGPB interaction for total yield revealed that the combination of ‘Lucky’ with *B. subtilis* produced the highest yield among all treatments (32.6 t ha^−1^), exceeding both the cultivar average and the *B.* subtilis main effect mean, suggesting a potential synergistic interaction under the experimental conditions (**Figure 2**). These results indicate that, under the conditions of this study, ‘Lucky’ showed superior yield-related traits, and *B. subtilis* was associated with enhanced reproductive performance. The significant cultivar × PGPB interaction for total yield further suggests the importance of selecting compatible cultivar-strain combinations to maximize cauliflower productivity.

**Table 2:** Effect of PGPB on days to curd appearance, days to first harvest, curd diameter, curd fresh weight, curd dry weight and total yield of cauliflower cultivars.

Cultivar (C)	Days to curd appearance	Days to first harvest	Curd diameter (cm)	Curd fresh weight (kg)	Curd dry weight (kg)	total yield (t ha^-1^)
Snow Drift	66.6 ± 3.8c	102.0 ± 7.0c	23.0 ± 1.5c	0.87 ± 0.18c	0.09 ± 0.02b	24.1 ± 4.5c
Lucky	73.6 ± 6.2a	108.5 ± 5.1a	31.0 ± 1.7a	1.11 ± 0.12a	0.11 ± 0.02a	29.8 ± 4.7a
White Beauty	70.5 ± 5.2b	104.6 ± 6.3b	28.0 ± 1.3b	1.00 ± 0.14b	0.10 ± 0.01ab	26.2 ± 4.7b
**LSD _(p≤ 0.01)_**	**2.70**	**1.70**	**0.70**	**0.10**	**0.10**	**2.50**
*Bacillus* spp. (BS)						
B0	65.2 ± 3.8d	101.5 ± 5.3c	25.5 ± 1.3c	0.85 ± 0.12d	0.09 ± 0.01c	23.1 ± 3.1d
BP	68.3 ± 2.9c	103.0 ± 6.8c	26.8 ± 1.5b	0.96 ± 0.15c	0.10 ± 0.01b	25.0 ± 3.7c
BT	71.7 ± 5.8b	105.8 ± 5.5b	28.2 ± 1.5a	1.01 ± 0.20b	0.10 ± 0.01b	27.8 ± 3.6b
BS	75.6 ± 7.1a	109.8 ± 4.3a	28.7 ± 1.4a	1.15 ± 0.09a	0.12 ± 0.01a	31.0 ± 4.5a
**LSD _(p≤ 0.01)_**	**3.10**	**1.90**	**0.80**	**0.10**	**0.10**	**2.90**
**C × BS**	* **Figure 2**	**NS**	**NS**	**NS**	**NS**	*Figure 2

B0, Distilled water; BP, *B. pumilus*; BT, *B. thuringiensis*; BS, *B. subtilis*; Values are means of three replications (blocks) ± standard deviation (SD). Within each plot, five individual plants were sampled and averaged to obtain one value per plot before statistical analysis. Means followed by different lowercase letters within a column are significantly different at p ≤ 0.05 according to the Least Significant Difference (LSD) test. NS = non-significant; * = significant at p ≤ 0.05.

Bold values in the LSD row are formatted for visual emphasis only and do not represent a statistical comparison.

### 3.3 Biochemical and quality parameters

**Figure 3:**
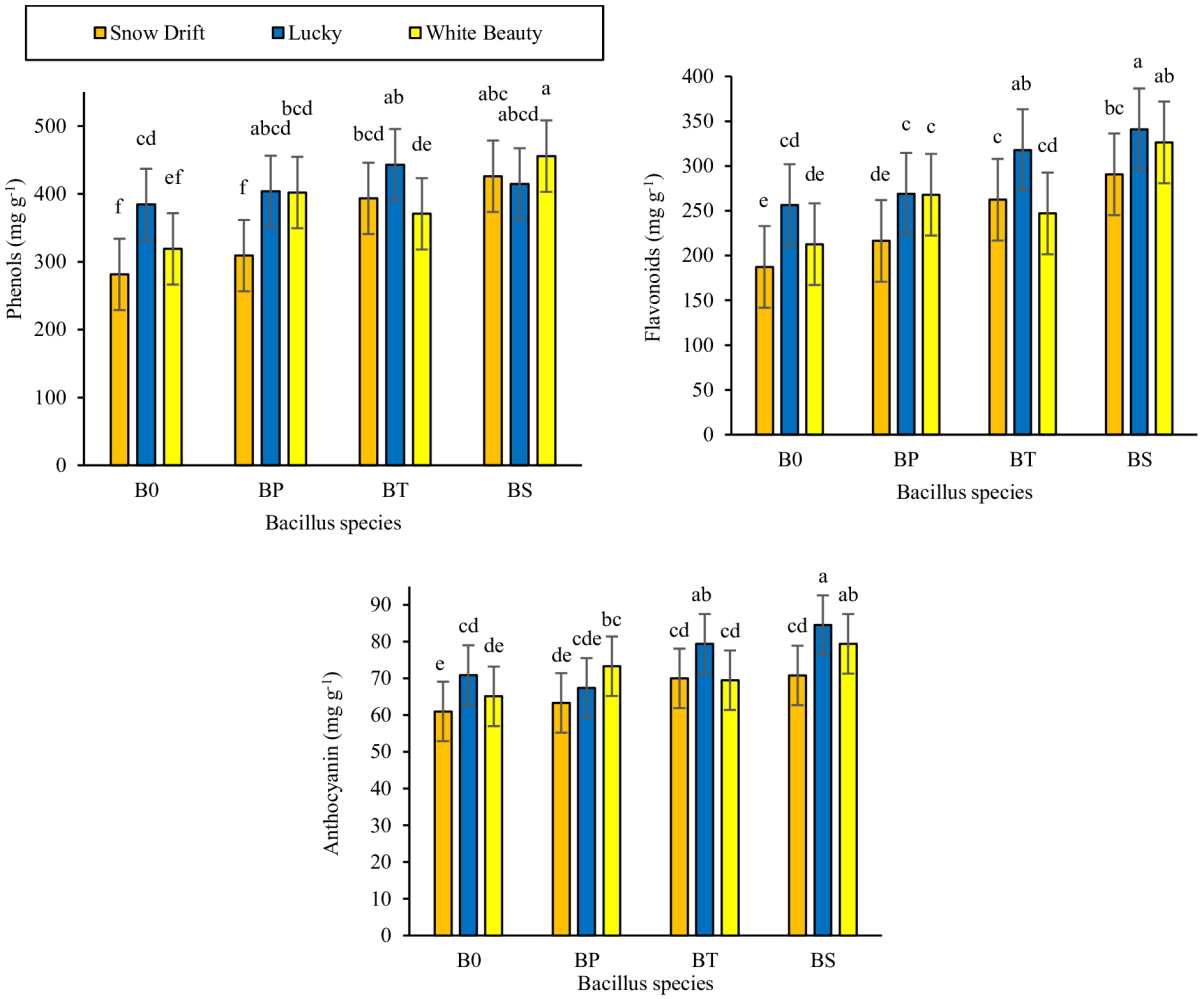
Effects of *Bacillus species* (B0, Distilled water; BP, *Bacillus pumilus*; BT, *Bacillus thuringiensis*; BS, *Bacillus subtilis*) on total phenols, flavonoids and anthocyanin content of cauliflower cultivars (Snow drift, Lucky and White beauty). Different lowercase letters indicate significant differences among treatments (LSD test, p<0.05).

Data pertaining to biochemical parameters in **Table 3** reveals that there was significant variation among cauliflower cultivars and in response to PGPB inoculation, with significant cultivar × PGPB interactions for total phenolic and anthocyanin content, while flavonoids and carotenoids exhibited non-significant interactions. Bioactive compound accumulation varied significantly among cauliflower cultivars and in response to PGPB inoculation (**Table 3**). Among cultivars, ‘Lucky’ consistently showed the highest values for total phenolics, flavonoids, anthocyanins, and carotenoids, followed by ‘White Beauty’, while ‘Snow Drift’ showed the lowest values across all parameters. Across all cultivars, PGPB inoculation increased bioactive compound accumulation compared to the control, with *B. subtilis* associated with the highest values, followed by *B. thuringiensis.* The significant cultivar × PGPB interactions for total phenolic, flavonoid, and anthocyanin content revealed that the highest flavonoid and anthocyanin content were observed in cultivar ‘Lucky’ treated with *B. subtilis*, while the maximum phenolic content was recorded in cultivar ‘White Beauty’ inoculated with *B. subtilis*, followed by cultivar ‘Lucky’ treated with *B. thuringiensis* (these two were statistically similar) (**Figure 3**). These results suggest that, under the conditions of this study, ‘Lucky’ showed superior accumulation of bioactive compounds, and *B. subtilis* was associated with enhanced nutritional quality. The significant interactions for phenolic and anthocyanin content further underscore the importance of selecting compatible cultivar-strain combinations to maximize the nutraceutical value of cauliflower.

**Table 3:** Effect of PGPB on curd dry weight, total phenols, flavonoids, anthocyanin and carotenoids contents of cauliflower cultivars.

Cultivar (C)	Total phenols (mg g^-1^)	Flavonoids (mg g^-1^)	Anthocyanin (mg g^-1^)	Carotenoids (mg g^-1^)
Snow Drift	352.3 ± 68.4c	239.0 ± 46.1c	66.0 ± 6.9c	2.6 ± 0.30b
Lucky	413.0 ± 35.2a	295.9 ± 51.1a	75.5 ± 8.2a	3.0 ± 0.31a
White Beauty	380.9 ± 58.9b	263.4 ± 49.9b	71.4 ± 6.7b	2.9 ± 0.28a
**LSD _(p≤ 0.01)_**	23.50	22.70	3.30	0.16
Bacillus spp. (BS)				
B0	328.0 ± 48.5c	218.7 ± 35.5c	64.7 ± 5.5d	2.6 ± 0.24b
BP	371.5 ± 56.5b	251.0 ± 34.5b	68.7 ± 7.0c	2.7 ± 0.26b
BT	397.8 ± 36.9b	275.5 ± 38.6b	72.6 ± 6.5b	2.9 ± 0.24a
BS	431.0 ± 39.3a	319.2 ± 48.5a	77.9 ± 7.1a	3.1 ± 0.28a
**LSD _(p≤ 0.01)_**	27.10	26.30	3.70	0.19
**C × BS**	*Figure 3	*Figure 3	*Figure 3	NS

B0, Distilled water; BP, *B. pumilus*; BT, *B. thuringiensis*; BS, *B. subtilis*; Values are means of three replications (blocks) ± standard deviation (SD). Within each plot, five individual plants were sampled and averaged to obtain one value per plot before statistical analysis. Means followed by different lowercase letters within a column are significantly different at p ≤ 0.05 according to the Least Significant Difference (LSD) test. NS, non-significant; *significant at p ≤ 0.05.

Bold values in the LSD row are formatted for visual emphasis only and do not represent a statistical comparison.

## 4 Discussion

The superior performance of ‘Lucky’ cultivar across all vegetative parameters—including plant height, leaf number, leaf area, chlorophyll content, and survival percentage—may be related to its inherent genetic potential and adaptability to the prevailing environmental conditions, supporting previous findings that genetic composition plays a fundamental role in determining plant growth traits in cauliflower (Sharma et al., 2017; Wang et al., 2015). These genetic differences among cultivars significantly influence leaf area development, photosynthetic capacity, and nutrient uptake efficiency, with cultivars like ‘Lucky’ demonstrating enhanced vegetative growth potential that directly contributes to improved productivity and quality (Islam et al., 2018; Ali et al., 2016). Foundational studies have established that PGPB can improve crop performance through nutrient solubilization, phytohormone production, and stress mitigation (Vessey, 2003; Lugtenberg and Kamilova, 2009; Glick, 2012). A recent review by Backer et al. (2018) emphasized that the effectiveness of PGPB is highly dependent on environmental conditions and plant genotype, a consideration directly relevant to the interpretation of the present results. The significant enhancement of all vegetative parameters by *B. subtilis* inoculation aligns with extensive research demonstrating the multifaceted mechanisms of PGPB, including improved nutrient uptake and solubilization, phytohormone production (particularly auxins and gibberellins), associated with enhanced photosynthetic pigment synthesis, and increased resistance to both biotic and abiotic stresses (Ekinci et al., 2014; Ahemad, 2015). Specifically, *B. subtilis* has been shown to increase plant height in various crops, including maize (Arslan and Bulut, 2023) and cauliflower (Kaushal and Kaushal, 2013), while also enhancing leaf production through organic acid synthesis and improved plant development parameters (Sunar et al., 2015). The increased leaf area observed in PGPB-treated plants results from improved root architecture and subsequent leaf expansion, leading to greater biomass accumulation, consistent with findings by Jayamini et al. (2021), who reported synergistic effects of PGPB application under various environmental conditions.

The observed increase in chlorophyll content in *Bacillus*-treated plants is consistent with previous reports that PGPB can improve plant nutrient status, particularly nitrogen and magnesium uptake, modulate plant hormone levels, and ameliorate abiotic stress (Indiragandhi et al., 2008; Pii et al., 2015; Cheng et al., 2021). Similarly, Ekinci et al. (2014) reported increased chlorophyll content in cauliflower transplants following PGPB inoculation, which aligns with our findings. The improved survival percentage observed in the ‘Lucky’ cultivar and *B. subtilis*-treated plants may be related to enhanced stress tolerance and overall plant vigor. Previous studies have shown that PGPB can improve cauliflower yield and biochemical parameters when combined with organic amendments and nutrient sources (Širić et al., 2022; Thakur et al., 2018; Yıldırım, 2022), and may also affect pest biology and plant longevity (Jahan et al., 2013). However, the specific mechanisms underlying these responses were not directly investigated in the present study. Taken together, the results suggest a positive effect of combining the ‘Lucky’ cultivar with *B. subtilis* on vegetative growth parameters in cauliflower. This effect may be attributed to complementary contributions from host genetic potential and microbial activity, though the specific mechanisms remain to be fully characterized.

Collectively, these results suggest that, under the conditions of this study, the combination of the ‘Lucky’ cultivar with *B. subtilis* was associated with enhanced vegetative growth in cauliflower. The superior reproductive and yield performance of the ‘Lucky’ cultivar—including delayed curd appearance and harvest, larger curd diameter, higher fresh and dry weight, and greater total yield—may be related to its phenotypic characteristics. Previous research has suggested that genetic factors, such as temperature-sensitive QTL regions on chromosomes C06 and C09 (Hasan et al., 2016), as well as enhanced photosynthetic capacity associated with greater leaf area and chlorophyll content (Jindal and Thakur, 2003), can influence curd development and photosynthate translocation. However, as no genetic or molecular analyses were conducted in the present study, these explanations remain speculative and are drawn from the literature rather than directly supported by our data. Consistent with previous reports, genetic differences among cauliflower cultivars have been shown to influence maturity timing (Hasan et al., 2016; Gurusamy, 1999), curd diameter (Cebula et al., 2005), fresh and dry weight accumulation (Yu-Hao et al., 2026; Islam et al., 2018), and overall yield potential (Ahmad et al., 2004). Our findings are in line with these studies, as ‘Lucky’ showed superior performance for these traits under the prevailing environmental conditions. The observed increases in yield parameters following *B. subtilis* inoculation, particularly the 33.3% increase in curd dry weight and the combination with ‘Lucky’ producing the highest total yield (32.6 t ha^−1^), are consistent with extensive research documenting PGPB-mediated improvements in cauliflower productivity. Recent studies have emphasized that PGPB effects are highly dependent on environmental conditions and plant genotype (Wahab et al., 2025; Tariq et al., 2025). Previous studies have reported mechanisms such as enhanced nutrient uptake and solubilization, phytohormone production (particularly auxins and gibberellins), improved root development, and delayed senescence (Ekinci et al., 2014; Thakur et al., 2018; Kaushal and Kaushal, 2013; Yıldırım, 2022). Fanai et al. (2024) highlighted the variability of PGPB performance under field conditions, which is directly relevant to the interpretation of the present results. While our study did not directly measure these mechanisms, our observations are consistent with these reported effects.

The delayed curd appearance and harvest observed in PGPB-treated plants, while potentially extending the growing period, was associated with superior curd quality and weight. This finding is consistent with reports that *B. subtilis* can delay senescence while promoting long-term agricultural benefits (Lastochkina et al., 2021; Saharan and Nehra, 2011), and that PGPB application may reduce chemical fertilizer requirements while improving crop responses in vegetable production (Seymen et al., 2019). The significant cultivar × PGPB interaction for total yield observed in this study underscores the potential importance of cultivar-strain compatibility, supporting previous reports of associations between cauliflower genotypes and specific bacterial strains (Mukherjee, 2022). Factors such as plant density (Salter and James, 1975), environmental conditions (Booij, 1987), and vitamin-mediated interactions (Palacios et al., 2014) may further modulate these responses, though these were not tested in our experiment. These results suggest a positive effect of combining the ‘Lucky’ cultivar with *B. subtilis* on cauliflower yield and quality parameters. While these effects may be mediated by complementary mechanisms involving host genetics, nutrient dynamics, and microbial activity, the present study does not provide direct evidence for these pathways. Further investigation is required to establish causality and to assess the reproducibility of these findings under diverse field conditions.

Collectively, these results suggest that, under the conditions of this study, the combination of the ‘Lucky’ cultivar with *B. subtilis* was associated with improved cauliflower yield and quality. The superior biochemical performance of the ‘Lucky’ cultivar—including higher total phenolic, flavonoid, anthocyanin, and carotenoid content—is consistent with previous reports of genetic variation in secondary metabolite production in cauliflower (González-Aguilar et al., 2010; Panche et al., 2016; Scalzo et al., 2008). Previous studies have shown that specific cultivars may exhibit enhanced phenolic profiles associated with resistance to oxidative stress and improved plant health (Giuffrida et al., 2018). Genetic differences in the flavonoid biosynthesis pathway (Andini et al., 2019), anthocyanin metabolic regulation (Mazza and Miniati, 2018), and carotenoid biosynthetic capacity (Rodriguez-Amaya, 2016) have been reported to contribute to cultivar variation, and breeding programs have increasingly targeted carotenoid enhancement for nutritional and visual appeal, as seen in cultivars such as ‘Cheddar’ and ‘Verde di Macerata’ (Diamante et al., 2021). However, as no genetic or molecular analyses were conducted in the present study, these explanations are drawn from the literature and remain speculative with respect to our specific findings.

The observed increases in bioactive compounds following *B. subtilis* inoculation, particularly the 46% increase in flavonoid content, are consistent with previous research documenting PGPB-mediated improvements in cauliflower nutritional quality. Previous studies have reported mechanisms such as bacterial-induced activation of the phenylpropanoid pathway, enhanced secondary metabolite biosynthesis, and improved plant stress responses (Ekinci et al., 2014; Thakur et al., 2018; Yıldırım, 2022). While our study did not directly measure these mechanisms, our observations align with these reported effects. The significant cultivar × PGPB interactions for total phenolic and anthocyanin content observed in this study suggest the potential importance of cultivar-strain compatibility for maximizing nutraceutical value, though this finding requires further validation.

The significant cultivar × PGPB interactions for total phenolic and anthocyanin content underscore the importance of cultivar-strain compatibility, with the ‘Lucky’ × *B. subtilis* combination producing the highest phenolic and anthocyanin accumulation, supporting the critical role of bacterial- and root-excreted compounds in mediating multipartite cross-talk between plants and beneficial microbes (Zhou et al., 2016; Palacios et al., 2014). Environmental and management factors further modulate these responses, including irrigation and plant density which increase phenolic substances (Kałużewicz et al., 2017), nutrient deficiencies such as nitrogen or phosphorus limitation that can enhance anthocyanin production (Hodges and Nozzolillo, 1996), and organic matter application combined with chemical fertilizers that indirectly influence bioactive compound accumulation (Tocci et al., 2018). Additionally, PGPB-mediated improvements in plant health through defense mechanism activation (Pannecoucque and Höfte, 2009; Choure and Dubey, 2012) and enhanced nutrient uptake (Kaushal and Kaushal, 2013) contribute to the observed increases in secondary metabolite production. Moreover, bio-inoculation strategies have been shown to influence plant physiological responses and hormone-mediated effects (Ipparraguirre et al., 2025), which may partly explain the observed improvements in reproductive performance. These findings are consistent with the hypothesis that combining genetically superior cultivars such as ‘Lucky’ with effective PGPB strains including *B. subtilis* may improve the nutraceutical value of cauliflower. The proposed roles of genetic potential for secondary metabolite biosynthesis and microbial facilitation of biochemical pathways require further investigation through targeted mechanistic studies.

## 5 Conclusion

The results of this study suggest that both cultivar selection and PGPB inoculation influenced the growth, yield, and quality attributes of cauliflower under the conditions evaluated. Among the three cultivars tested, ‘Lucky’ showed the highest values for vegetative growth, reproductive performance, and bioactive compound accumulation, indicating phenotypic variability among the tested cultivars. Among the PGPB strains tested, *B. subtilis* was associated with the highest mean values for vegetative parameters (plant height, leaf number, leaf area, chlorophyll content, and survival), reproductive attributes (curd dimensions, fresh and dry biomass, and total yield), and bioactive compounds (phenolics, flavonoids, anthocyanins, and carotenoids).

The combination of the ‘Lucky’ cultivar with *B. subtilis* showed higher performance for several traits compared to other cultivar–strain combinations. However, this study was conducted at a single location and during one growing season, with a limited number of cultivars and bacterial strains. Therefore, further validation under different environmental conditions, across multiple growing seasons, and with a wider range of genotypes and PGPB strains is required before practical recommendations can be made for agricultural production. These findings contribute preliminary insights for the potential development of cultivar-specific bio-inoculant strategies in sustainable vegetable production.

## Data Availability

The original contributions presented in the study are included in the article/supplementary material. Further inquiries can be directed to the corresponding authors.
